# Signal Transduction for TNFα-Induced Type II SOCS Expression and Its Functional Implication in Growth Hormone Resistance in Carp Hepatocytes

**DOI:** 10.3389/fendo.2020.00020

**Published:** 2020-01-30

**Authors:** Xue Jiang, Mulan He, Jin Bai, Chi Bun Chan, Anderson O. L. Wong

**Affiliations:** School of Biological Sciences, The University of Hong Kong, Hong Kong, China

**Keywords:** TNFα, SOCS, CISH, GH, IGF, signal transduction, hepatocytes, grass carp

## Abstract

In mammals, local production of tumor necrosis factor α (TNFα) inhibits growth hormone (GH)-induced IGF-I expression at tissue level and contributes to GH resistance caused by sepsis/endotoxemia and inflammation. Although the loss of GH responsiveness can be mediated by a parallel rise in SOCS expression, the signaling mechanisms for TNFα-induced SOCS expression at the hepatic level have not been characterized and the comparative aspects of the phenomenon, especially in lower vertebrates, are still unknown. Recently, type II SOCS, including SOCS1-3 and CISH, have been cloned in grass carp and shown to act as the feedback repressors for GH signaling via JAK_2_/STAT_5_ pathway. To shed light on the mechanisms for TNFα-induced GH resistance in fish model, grass carp TNFα was cloned and confirmed to be a single-copy gene expressed in various tissues including the liver. In carp hepatocytes, incubation with the endotoxin LPS induced TNFα expression with parallel rises in SOCS1-3 and CISH mRNA levels. Similar to LPS, TNFα treatment could block GH-induced IGF-I/-II mRNA expression and elevate SOCS1, SOCS3, and CISH transcript levels. However, TNFα was not effective in altering SOCS2 expression. In parallel experiment, LPS blockade of IGF-I/-II signals caused by GH could be partially reverted by TNFα receptor antagonism. At hepatocyte level, TNFα induction also triggered rapid phosphorylation of IκBα, MEK_1/2_, ERK_1/2_, MKK_3/6_, P_38_^MAPK^, Akt, JAK_2_, and STAT_1,3,5_, and TNFα-induced SOCS1, SOCS3, and CISH mRNA expression could be negated by inhibiting the IKK/NFκB, MAPK, PI3K/Akt, and JAK/STAT cascades. Our findings, as a whole, suggest that local production of TNFα may interfere with IGF-I/-II induction by GH in the carp liver by up-regulation of SOCS1, SOCS3, and CISH via IKK/NFκB, MAPK, PI3K/Akt, and JAK/STAT-dependent mechanisms, which may contribute to GH resistance induced by endotoxin in carp species.

## Introduction

Tumor necrosis factor α (TNFα) is a member of the proinflammatory cytokines and plays a key role in regulating immune cell activation/migration, cell proliferation/apoptosis, angiogenesis, and insulin resistance/growth impairment caused by chronic stress/infection (1). TNFα is also involved in diseases/ pathological conditions related to immune disorder, e.g., rheumatoid arthritis, ankylosing spondylitis, psoriasis, and inflammatory bowel disease (2). TNFα, together with other proinflammatory cytokines (e.g., IL-1β and IL-6), can also act as local mediators for GH resistance induced by sepsis/endotoxemia or chronic inflammation (3–5). During the process, serum GH can be elevated with current drops in circulating IGF-I (6) and its production at tissue level (e.g., in muscle and liver) (4, 6). Meanwhile, a loss in hepatic responsiveness for GH-induced JAK_2_/STAT_5_ signaling and IGF-I gene expression has also been reported, e.g., in rat hepatocytes (7) and CWSV-1 hepatic cells (8, 9). GH resistance triggered by local production of cytokines can be attributed to a drop in GH receptor (GHR) expression via inhibition on GHR promoter activity (10–12) with parallel activation of SOCS expression (e.g., SOCS1, SOCS3, and CISH) (3, 12, 13), which can lead to failure in linear growth during childhood development (14) as well as muscle wasting, poor wound healing, cachexia, hepatic steatosis and insulin resistance in adult stage (14, 15). Although modulation of GH responsiveness by cytokines represents a major mechanism for the functional crosstalk of the immune system with somatotropic axis (16), the studies in this area are restricted to mammalian models and the comparative aspects of the phenomenon, especially in lower vertebrates, are still unknown.

SOCS proteins are feedback inhibitors for cytokine signaling and their inhibitory effects are mediated through interference of the JAK/STAT pathway functionally coupled with cytokine receptors (17). They are widely expressed at the tissue level (18) and can be induced by microbial/viral infection (19, 20) via local production of cytokines (e.g., IL-4 and IFNγ) (21, 22). By limiting the duration/magnitude of cytokine signaling, SOCS expression can prevent hyper activation of immune responses and bring the system back to a homeostatic state (23), and dysregulation of SOCS can be associated with autoimmune diseases, chronic inflammation and carcinogenesis (24, 25). At present, at least 10 members of SOCS family, including SOCS1-9 and CISH, have been identified. They can be classified into type I (SOCS4-7 and SOCS5b-9) and type II subfamily (SOCS1-3 and CISH) with the type II SOCS closely related to the ancestral lineage found in invertebrates (26). Members of SOCS family all share a common structural organization with a SH2 domain in the central core followed by a SOCS box in the C-terminal. The SH2 domain can bind to the phosphotyrosine residues in cytokine receptors or activated JAKs and is essential for target recognition, and presumably, can also interfere with receptor coupling with the JAK/STAT pathway (27). The SOCS box can trigger E3 ubiquitin ligase assembly to induce ubiquitination and proteosomal degradation of the signaling complex formed by JAK and cytokine receptor (28). An additional motif, namely the kinase inhibitory region, has been reported in SOCS1 and SOCS3 but not in other SOCS members (29), which can act as a pseudo-substrate domain to inhibit JAK activity (30). In different cell types (e.g., hepatocytes and macrophages), TNFα is involved in SOCS expression (e.g., SOCS3) induced by endotoxin/inflammation (31–33). SOCS expression, in agreement with its role as feedback signals for cytokines, has been reported to inhibit TNFα actions, e.g., SOCS1 over-expression can block TNFα-induced caspase activity and apoptosis in cardiomyocyte (34) and fibroblast culture prepared from rodents (35). Although TNFα receptors, namely TNFR1 and TNFR2, are known to mediate TNFα actions via activation of the IKK/NFκB, JAK/STAT, MAPK, and TRADD/caspase pathways (36–38), there is a general lack of information on the mechanisms for TNFα-induced SOCS expression. Except for two reports, one in 3T3 cells (39) and the other in smooth muscle cells (40), showing the involvement of MAPK signaling in TNFα-induced SOCS3 expression, not much is known for the signal transduction for SOCS induction by TNFα. Of note, the post-receptor signaling for the corresponding responses occurred at the hepatic level, especially during GH resistance induced by endotoxin, has not be examined and represents an important topic for further investigation.

Recently, the key members of type II SOCS, SOCS1-3 and CISH, have been cloned in grass carp and confirmed to be inducible by GH and serve as the feedback repressors for GH signaling via the JAK_2_/STAT_5_ pathway with inhibitory effects on both basal and GH-induced IGF-I gene transcription at the hepatic level (41). In fish models, cytokine induction by microbial/viral infection [e.g., TNFα and interleukins in carp species (42, 43)] and IGF-I modulation by cytokines [e.g., TNFα) have been reported [e.g., in immune cells/tissues of trout and sea bass, see (44) for a recent review]. In turbot, bacterial infection is also effective in increasing SOCS3 mRNA levels in various tissues including the liver and SOCS3 over-expression can inhibit TNFα and IL-1β expression in macrophages isolated from the head kidney (45). These findings not only suggest that the role of SOCS as feedback inhibitors to fine tone the immune responses is well-conserved from fish to mammals, but also raise the possibility that SOCS expression can form a functional link between the somatotropic axis and immune system in fish models. Since the spreading of bacterial/viral infection is a major concern in fish culture, which will also adversely affect the growth performance of culture species, study has been initiated in our laboratory to examine the mechanisms for GH resistance in fish model induced by endotoxin of bacteria origin. Grass carp was chosen as the animal model as it is a representative of the carp family and by itself constitutes a major aquacultural output in Asian countries (~4.6 million tons per year and account for 15.6% of global finfish production) (FAO yearbook of Fishery and Aquaculture Statistics 2011). In this study, the questions to be addressed are: (i) Can TNFα be induced by lipopolysaccharide (LPS, an endotoxin in gram-negative bacteria) and contribute to GH resistance in the carp liver along with the corresponding responses in SOCS? (ii) What are the subtypes of SOCS members induced by LPS at the hepatic level mediated by local production of TNFα signal? (iii) What are the signal transduction mechanisms involved in TNFα-induced SOCS expression which may contribute to GH resistance? As a first step, TNFα was cloned in grass carp and its tissue expression, especially in the liver, was confirmed by RT-PCR and LC/MS/MS. Using primary culture of carp hepatocytes, TNFα expression was examined with exposure to LPS and correlated to the corresponding changes in SOCS1-3 and CISH mRNA levels using real-time PCR. To evaluate if GH resistance could occur in the carp liver by TNFα signal induced by LPS, GH-induced IGF-I and -II mRNA expression in carp hepatocytes were tested with co-treatment of LPS or TNFα, respectively. Using a combination of pharmacological approach coupled with immunoblotting of the phosphorylation status of respective signaling targets/kinases, the signal transduction mechanisms for SOCS expression induced by TNFα were also elucidated at the hepatic level. Our studies, as a whole, have shed light on the mechanisms involved in GH resistance induced by endotoxin occurring in the liver of a fish model.

## Materials and Methods

### Animals and Test Substances

One-year-old grass carp (*Ctenopharyngodon idellus*) were purchased from local wholesale markets and maintained in well-aerated 200 L aquaria at 18°C under at 12L:12D photoperiod. Since the carp at this stage was pre-pubertal and sexual dimorphism was not apparent, fish of mixed sexes were used for tissue sampling and hepatocyte preparation. During the process, the fish was killed by anesthesia in 0.05% MS222 (Sigma, St. Louis, MO) followed by spinosectomy according to the protocol approved by the Committee for Animal Use in Teaching and Research at the University of Hong Kong (Hong Kong). Human TNFα and bovine GH were was obtained from R&D Systems (Minnesota, MN). The IκB kinase (IKK) inhibitor N-(4-Pyrrolidin-1-yl-piperidin-1-yl)-[4-(4-benzo[b]thiophen-2-yl-pyrimidinylamino) phenyl]carboamide (IKK16) and NF-κB activation blocker N-[(Phenyl methoxy)carbonyl]-L-leucyl-N-[(1S)-1-formyl-3-methylbutyl]-L-leucinamide (MG132) were acquired from TOCRIS (Eillsville, MO). The JAK_2_ inhibitor 1,2,3,4,5,6-hexabromocyclohexane (Hex), STAT_1_ inhibitor Fludarabine (FA), STAT_3_ inhibitor Ethyl-1-(4-cyano-2,3,5,6-tetrafluorophenyl)-6,7,8-trifluoro-4-oxo-1,4-dihydroquinoline-3-carboxylate (ETDDC), STAT_5_ inhibitor N1-(11H-Indolo[3,2-c] quinolin-6-yl)-N2,N2-dimethylethane-1,2-diamine (IQDMA), MEK_1/2_ inhibitor U0126, ERK_1/2_ inhibitor SCH 772984, P_8_^MAPK^ inhibitor PD169316, PI3K inhibitor Ly294002, Akt inhibitor HIMOC and the endotoxin LPS were procured from Calbiochem (San Diego, CA). Test substances, except for TNFα and GH dissolved in double-distilled deionized water, were dissolved in DMSO and stored frozen at −80°C in small aliquots. On the day of experimentation, test substances were thawed on ice and diluted to appropriate levels with prewarmed medium 15 min prior to drug administration. The final dilutions of DMSO were always ≤ 0.1% and did not affect TNFα and SOCS mRNA expression in carp hepatocytes. In our studies, GH and TNFα of mammalian origin were used as the functional substitutes for the fish counterparts, as these mammalian proteins are commercially available and confirmed to be bioactive by previous reports in fish models [e.g., in carp (41) and rainbow trout (46, 47)].

### Molecular Cloning, Gene Copy Number, and Tissue Expression of TNFα

For molecular cloning of grass carp TNFα, total RNA was isolated from the carp liver with TRIZOL and subjected to 5′/3′ RACE using primers designed based on the conserved regions of TNFα reported in zebrafish. Sequence alignment, 3D protein modeling, and phylogenetic analysis were conducted using CLUSTAL-W (https://www.genome.jp/tools-bin/clustalw), SWISS-MODEL (https://swissmodel.expasy.org/) and MEGA 6.0 (https://www.megasoftware.net/), respectively. To deduce the gene copy number of TNFα, Southern blot was performed in genomic DNA isolated from the whole blood of grass carp as described previously (41). For tissue expression of TNFα, RT-PCR was conducted in selected tissues and brain areas using primers for carp TNFα (Forward primer: 5′-GCTTCACGCTCAACAAGTCTCA-3′; Reverse primer: 5′-AGCCTGGTCCTGGTTCACTCT-3′). PCR for TNFα expression was conducted with denaturation at 94°C for 3 min followed by 37 cycles of denaturation at 94°C for 30 s, annealing at 56°C for 30 s, and extension at 72°C for 30 s, and concluded with a final extension step at 72°C for 5 min. The authenticity of the PCR products obtained was confirmed by Southern blot using a DIG-labeled cDNA probe for carp TNFα and parallel PCR for β actin was used as an internal control. Using LC/MS/MS, protein expression of TNFα in the carp liver was also evaluated using a SCIEX TripleTOF-5600 system (AB SCIEX, Concord, ON, Canada) according to the standard protocol in our laboratory (48).

### TNFα and Type II SOCS mRNA Expression in Carp Hepatocytes

Primary culture of grass carp hepatocytes was prepared by collagenase digestion (41) and maintained in 24-well plates at a seeding density of ~0.7 × 10^6^ cells/ml/well. After drug treatment, total RNA was extracted from individual wells by TRIZOL, digested with DNase I, and reversely transcribed using Superscripts II (Invitrogen) with Oligo-dT as the primer. RT samples prepared were then subjected to real-time PCR for TNFα and type II SOCS mRNA measurement using a LightCycler 480 SYBR Green Master I Kit (Roche) with the RotorGene-Q qPCR System (Qiagen). In parallel experiments to evaluate GH resistance at the hepatic level, carp hepatocytes were exposed to GH with co-treatment of LPS or TNFα and the RT samples prepared were used for real-time PCR measurement of GHR and IGF-I/-II transcripts. Real-time PCR for respective gene targets, including TNFα, GHR, IGF-I/-II, and different members of type II SOCS, will be conducted according to the conditions described in Supplemental Table 1. In our studies, serial dilutions of plasmids with the ORF of the target genes were used as the qPCR standards for data calibration and parallel measurement of 18S RNA expression was used as the internal control. After the assays, the authenticity of PCR products was routinely confirmed by melting curve analysis.

### Western Blot of Signaling Targets and Kinases in Carp Hepatocytes

To test if TNFα can induce IKK/NF-κB, JAK/STAT, MAPK, and PI3K/Akt activation in the carp liver, Western blot was performed in cell lysate prepared from carp hepatocytes with TNFα treatment using the antibodies for phosphorylated form (“p-” form) and total protein (“t-” form) of IκBα (1:1,000; Santa Cruz), MEK_1/2_ (1:1,000; Cell Signaling), Erk_1/2_ (1:5,000; Sigma), P_38_^MAPK^ (1:1,000; Cell Signaling), Akt (1:1,000, Cell Signaling), JAK_2_ (1:1,000; Santa Cruz), STAT_1_ (1:1,000; Cell Signaling), STAT_3_ (1:1,000; Cell Signaling) and STAT_5_ (1:1,000; Cell Signaling), respectively. In these studies, parallel blotting of β actin with an Actin Ab-1 Kit (Calbiochem) was used as the internal control.

### Data Transformation and Statistical Analysis

For real-time PCR of TNFα, GHR, IGF-I/-II, SOCS1-3, and CISH mRNA levels, standard curves constructed with serial dilutions of plasmid DNA carrying the ORF of the respective gene targets with a dynamic range of ≥10^5^, amplification efficiency ≥0.98 and a correlation coefficient of ≥0.95 were used for data calibration with the RotorGene Q-Rex solfware (Qiagen). Given that the data for transcript expression of 18S RNA, the internal control for real-time PCR, did not exhibit significant changes in our studies, the raw data for target gene expression were simply transformed as a percentage of the mean value in the control group without drug treatment (as “%Ctrl”). For Western blot, except for the data of IκBα expressed as a ratio of IκBα signals over β actin expression in the same sample (due to a drop of total IκBα after treatment), the Western blot signals detected were quantitated by densitometry scanning in “arbitrary density unit” and expressed as the ratio of phosphorylated form over total protein of the same target. The normalized data were then expressed as fold increase compared to the control group without drug treatment (as “fold induction”). Data presented, expressed as mean ± SEM (*N* = 6), are pooled results from six independent experiments and analyzed using one-way (for dose-dependence/co-treatment studies with signaling inhibitors)/two-way ANOVA (for time course) followed by Newman–Keuls test. Differences between groups were considered as significant at *p* < 0.05.

## Results

### Molecular Cloning, Structural Characterization, and Tissue Expression of Grass Carp TNFα

**Figure 1:**
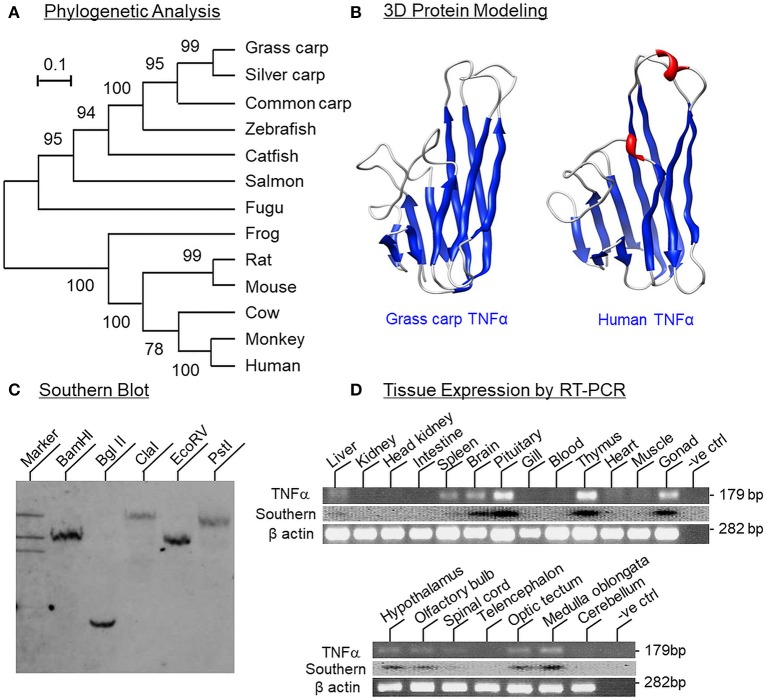
Phylogenetic analysis, protein modeling, genomic Southern and tissue distribution of grass carp TNFα. **(A)** Phylogenetic analysis of grass carp TNFα sequence with the neighbor-joining method using MEGA 6.0 with the corresponding sequences in other species. The scale bar represents the evolutionary distance and the values indicated in individual nodes of the guide tree are the percentage based on 1,000 bootstraps. **(B)** 3D Protein modeling of grass carp TNFα using the crystal structure of secreted form of human TNFα as the template with SWISS-MODEL program. The β sheets are presented in blue and α helixes in red. **(C)** Southern blot to evaluate the gene copy no of TNFα in the carp genome. Genomic DNA was isolated from grass carp and digested with restriction enzymes as indicated. After that, DNA samples were size-fractionated and hybridized with a DIG-labeled probe for carp TNFα. **(D)** Tissue expression profiling of TNFα using RT-PCR. Total RNA was isolated from selected tissues and brain areas and subjected to RT-PCR using primers specific for carp TNFα. The authenticity of PCR products was confirmed by PCR Southern and RT-PCR for β actin was used as the internal control.

To establish the structural identity of TNFα expressed in carp species, the full-length cDNA of grass carp TNFα (GenBank accession No. JQ040498) was cloned and found to be 1251 bp in size with a 720 bp ORF encoding a 239 a.a. protein (with deduced MW of ~26 kDa) flanking by a 126 bp 5′UTR and a 405 bp 3′UTR (Supplemental Figure 1). In the 3′URT, five AU-rich elements (ARE, “attta”) were also located in the region overlapping with three polyadenylation signals (“aaaaag” and “attaaa”) upstream of the poly(A) tail. Phylogenetic analysis of the nucleotide sequence obtained using the neighbor-joining method revealed that the newly cloned cDNA could be clustered within the clade of fish TNFα and closely related to the TNFα in carp species (Figure 1A). In the deduced a.a. sequence, the transmembrane domain and signature motif of the TNF family (IIIPDDGIYFVYSVSF) could be identified along with a TACE cleavage site (TL), three putative N-linked glycosylation sites (NXT/S) and two well-conserved Cys residues. Protein sequence alignment of grass carp TNFα with the corresponding sequences found in other species using CLUSTAL-W also confirms that the grass carp sequence is highly homologous to the TNFα reported in the carp family and to a lower extent when compared with the corresponding sequences in other fish species and tetrapods (Supplemental Figure 2). Of note, the twelve β sheets (namely β sheet 1–12) as a major structural characteristic of TNFα could also be identified in grass carp TNFα and the a.a. sequences of β sheet 5–6 (covering the signature motif of TNFα), β sheet 7–8, β sheet 9–10, and β sheet 12 were found to be highly conserved among different species. *In silico* protein modeling using the crystal structure of human TNFα as the template also showed that the 3D structure of the secreted form of carp TNFα (covering β sheet 3–12) could fit into a highly packed structure with 10 anti-parallel β strands arranged in a β-jellyroll topography (Figure 1B). The 3D model deduced for carp TNFα, especially the spatial arrangement and orientation of β sheets, was found to be highly comparable if not identical to that of the human counterpart except for the absence of two short helixes in the linker between β sheet 6 and 7.

To determine the gene copy number of TNFα, Southern blot was performed in DNA sample isolated from the whole blood of grass carp using a DIG-labeled probe for carp TNFα. After digestion of DNA sample with restriction enzymes including BamHI, Bgl II, Cla I, EcoR V, and Pst I, respectively, a single band was consistently observed in individual lanes of the Southern blot (Figure 1C), implying that TNFα is a single-copy gene in the carp genome. For tissue expression profiling of TNFα expression, RT-PCR was also conducted in selected tissues and brain areas in grass carp. As shown in Figure 1D, PCR signals for TNFα were found to be expressed at high levels in the pituitary, thymus and gonad, to a lower extent in the brain, spleen, and liver, and not detectable in the gills, heart, muscle, blood, kidney, intestine, and head kidney. In selected brain areas, TNFα signals were also detected in the hypothalamus, olfactory bulbs, optic tectum, medulla oblongata, and spinal cord, but not in the telencephalon and cerebellum. In these experiments, the PCR signals for β actin were consistently detected in all the samples examined, suggesting that the lack of TNFα signals due to RNA degradation was unlikely.

### TNFα Induction by LPS and Its Effects on IGF-I/-II and SOCS/CISH Expression in Carp Hepatocytes

**Figure 2:**
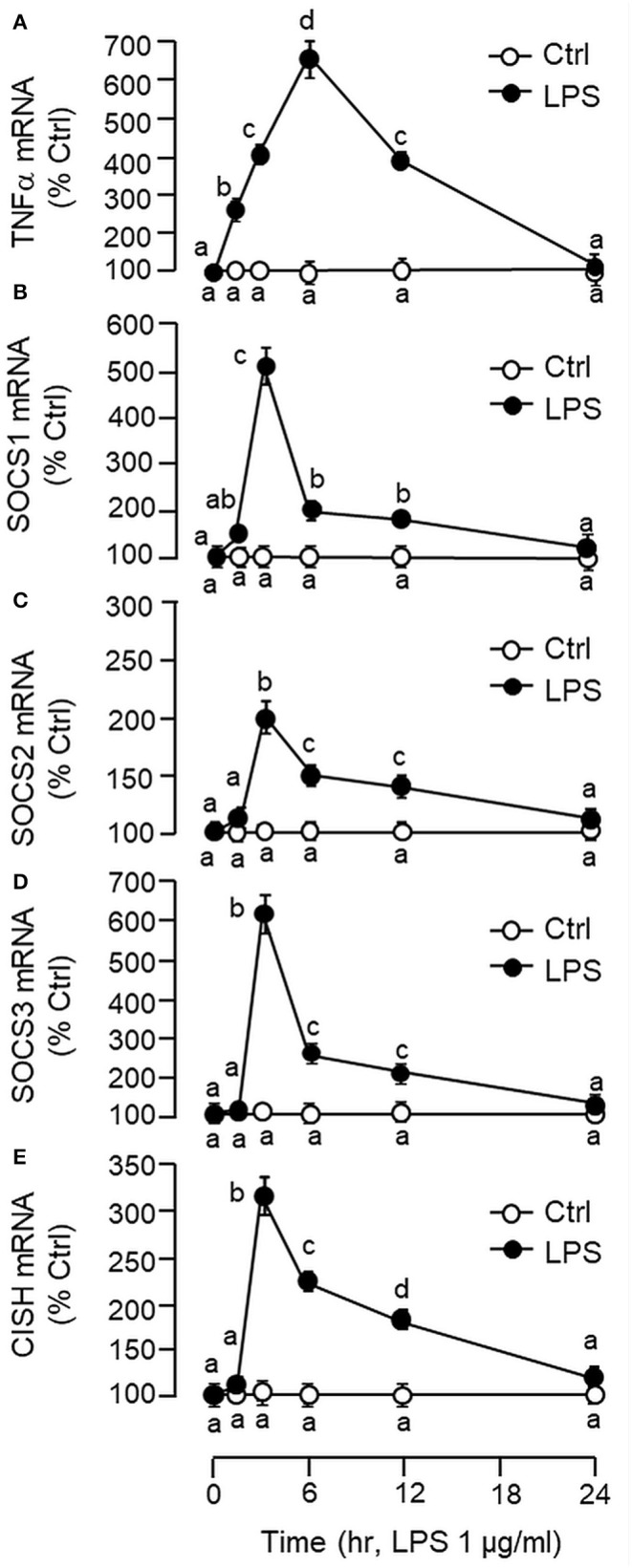
Effects of LPS on TNFα and type II SOCS expression in primary culture of carp hepatocytes. Hepatocytes were treated with LPS (1 μg/ml) for the duration as indicated. At the respective time points, total RNA was extracted from the cell culture, reversely transcribed and subjected to real-time PCR for TNFα **(A)**, SOCS1 **(B)**, SOCS2 **(C)**, SOCS3 **(D)**, and CISH mRNA measurement **(E)**. Data presented are expressed as mean ± SEM (*N* = 6) and experimental groups denoted by different letters represent a significant difference at *p* < 0.05 (ANOVA followed by Newman–Keuls Test).

Using LC/MS/MS, peptide fragments originated from TNFα were also detected in protein sample prepared from the carp liver with trypsin digestion (protein coverage by peptides identified: 92.7% for peptides with ≥75% confidence and 85.4% for peptides with 99% confidence), suggesting that the transcript signals of TNFα detected by RT-PCR can be properly translated into target protein in the carp liver (Supplemental Figure 3). To test for the presence of a functional TNFα/SOCS system at the hepatic level, primary culture of grass carp hepatocytes was challenged with the endotoxin LPS (1 μg/ml). In this case, a transient rise in TNFα mRNA levels with a peak response at 6 h was noted and the elevation in TNFα signals reduced gradually and returned to basal by the end of 24 h (Figure 2A). Meanwhile, LPS induction was also effective in increasing SOCS1-3 and CISH transcript expression with peak responses at 3 h (Figures 2B–E). Of note, a significant rise in TNFα signal (at 1.5 h) could be observed prior to the corresponding changes in SOC1-3 and CISH (at 3 h) and the peak responses of these type II SOCS also occurred before the “recovery phase” of TNFα expression (from 6 to 24 h).

**Figure 3:**
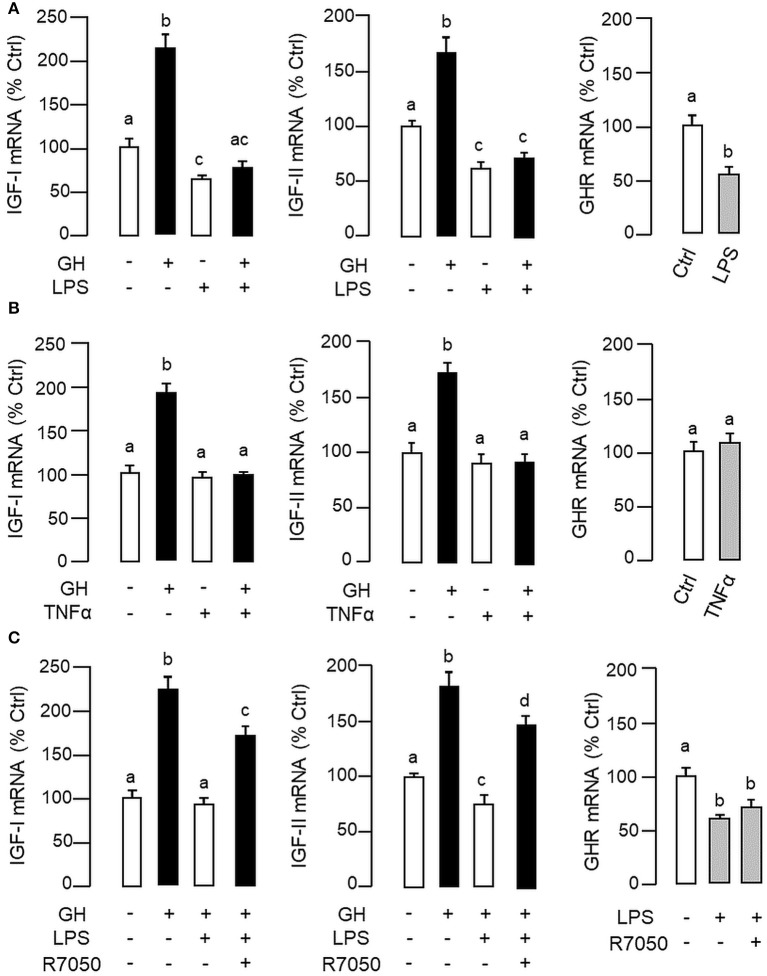
LPS and TNFα treatment on IGF-I/-II and GHR expression in carp hepatocytes. To examine the effects of LPS **(A)** and TNFα **(B)** on IGF-I/-II expression, hepatocyte culture was exposed to GH (500 ng/ml) with/without the co-treatment of LPS (1 μg/ml) or TNFα (100 ng/ml), respectively. In parallel experiments, hepatocytes were also treated with LPS (1 μg/ml) or TNFα alone (100 ng/ml) to study their effects on GHR expression. To shed light on the role of TNFα in LPS-induced GH resistance at the hepatic level, the effects of LPS (1 μg/ml) on IGF-I/-II responses induced by GH (500 ng/ml) and basal level of GHR expression were also tested with co-treatment of the TNFα receptor R7050 (10 μM) **(C)**. In these studies, the duration of drug treatment was fixed at 24 h. After treatment, total RNA was extracted for real-time PCR measurement of IGF-I/-II and GHR transcripts, respectively. Experimental groups denoted by different letters represent a significant difference at *P* < 0.05.

**Figure 4:**
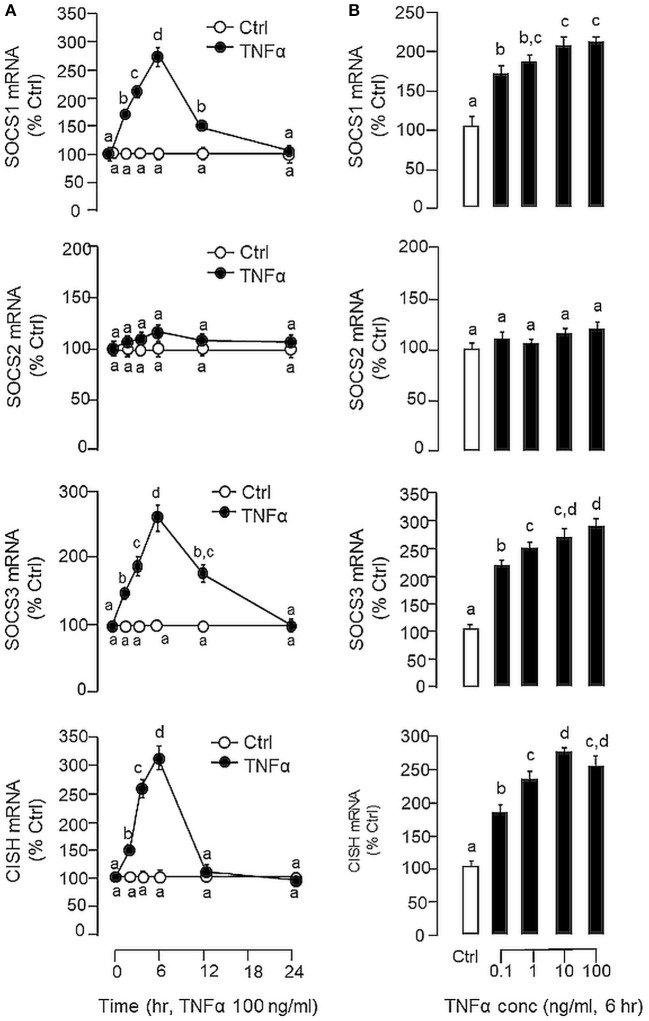
Effects of TNFα on type II SOCS expression in carp hepatocytes. **(A)** Time course and **(B)** dose dependence of TNFα treatment on SOCS1, SOCS2, SOCS3, and CISH expression. For time course study, hepatocytes were incubated with TNFα (100 ng/ml) for the duration as indicated up to 24 h. For dose dependence, cell culture were treated for 6 h with increasing concentrations of TNFα (0.1–100 ng/ml). After drug treatment, total RNA was extracted, reversely transcribed and subjected to real-time PCR for SOCS1, SOCS2, SOCS3, and CISH mRNA measurement, respectively. Experimental groups denoted by different letters represent a significant difference at *P* < 0.05.

In carp hepatocytes, GH induction (500 ng/ml) consistently induced IGF-I and -II mRNA expression and these stimulatory effects could be blocked by co-treatment with LPS (1 μg/ml, Figure 3A) or TNFα (100 ng/ml, Figure 3B), respectively. The blockade on GH-induced IGF-I and -II signals by LPS, however, was found to be partially reverted by the TNFα receptor antagonist R7050 (10 μM, Figure 3C). In these studies, LPS but not TNFα was also effective in reducing basal levels of IGF-I and -II as well as GHR mRNA expression (Figures 3A,B) but the inhibitory action on GHR by LPS was not affected by co-treatment with R7050 (Figure 3C). To test if the effects of LPS on SOCS expression could be mimicked by TNFα, time course and dose dependence studies were also conducted in carp hepatocytes with TNFα treatment. Interestingly, TNFα induction was found to up-regulate SOCS1, SOCS3, and CISH but not SOCS2 mRNA in a time- (Figure 4A) and dose-dependent manner (Figure 4B). Similar to the kinetics of LPS-induced TNFα mRNA expression, the gene expression of SOCS1, SOCS3, and CISH induced by TNFα reached their respective peaks at 6 h and gradually returned to basal by the end of 24 h.

### Signal Transduction for TNFα-Induced SOCS1, SOCS3, and CISH mRNA Expression at Hepatic Level

**Figure 5:**
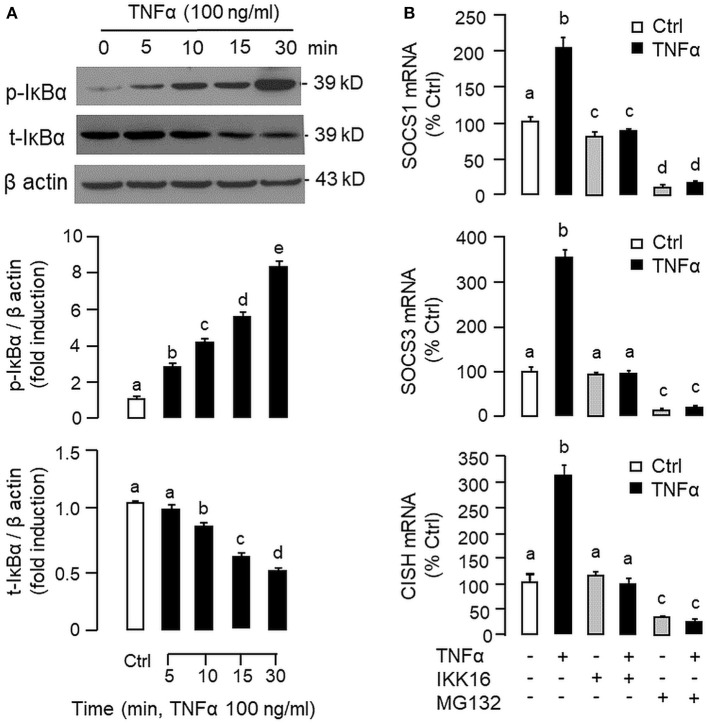
Functional role of IKK/NFκB pathway in TNFα-induced SOCS1, SOCS3, and CISH expression at the hepatic level. **(A)** TNFα treatment on IκBα phosphorylation in carp hepatocytes. Hepatocytes were treated with TNFα (100 ng/ml) for the duration as indicated. After that, cell lysate was prepared and subjected to Western blot with antibodies for the phosphorylated form (p-form) and total protein (t-form) of IκBα. In this study, parallel blotting of β actin was used as the internal control. The signals for the p-form and t-form of IκBα were quantified by densitometry scanning and normalized as a ratio of β actin expressed in the same sample. **(B)** Blocking IKK/NFκB pathway on TNFα-induced SOCS1, SOCS3, and CISH mRNA expression. Hepatocytes were treated for 6 h with TNFα (100 ng/ml) in the presence/absence of the IKK inhibitor IKK16 (10 μM) or NFκB inhibitor MG132 (10 μM). After that, total RNA was prepared and used for real-time PCR measurement of the respective transcripts. Experimental groups denoted by different letters represent a significant difference at *P* < 0.05.

**Figure 6:**
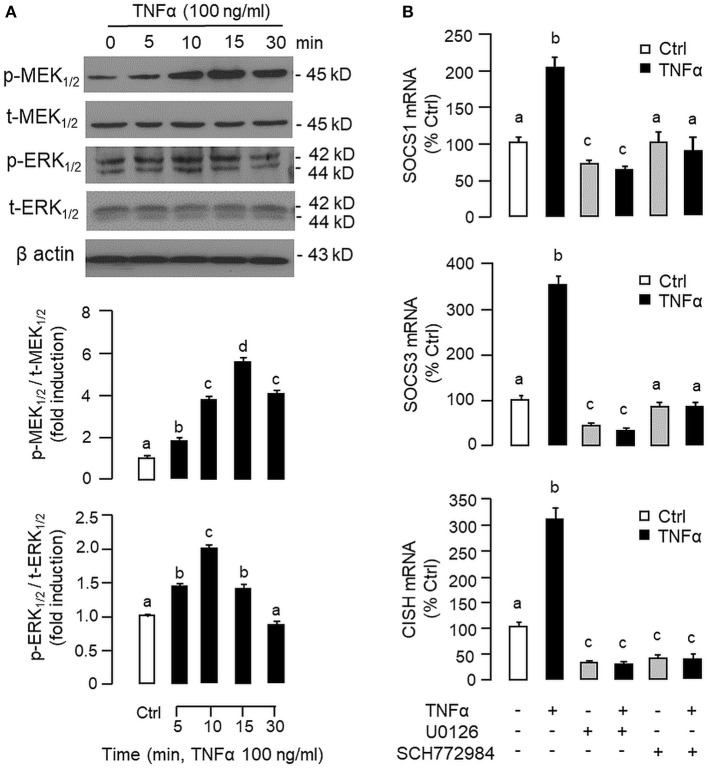
Functional role of MEK/ERK pathway in TNFα-induced SOCS1, SOCS3, and CISH expression at the hepatic level. **(A)** TNFα treatment on MEK_1/2_ and ERK_1/2_ phosphorylation in carp hepatocytes. Hepatocytes were treated with TNFα (100 ng/ml) for various durations up to 30 min. After that, cell lysate was prepared and used in Western blot with antibodies for the phosphorylate form (p-form) and total protein (t-form) of MEK_1/2_ and ERK_1/2_, respectively, and parallel blotting of β actin was used as the internal control. The signals for the two forms of protein targets were quantified by densitometry scanning and expressed as a ratio of the p-form/t-form in the same sample. **(B)** Blocking MEK/ERK pathway on TNFα-induced SOCS1, SOCS3, and CISH mRNA expression. Hepatocytes were treated for 6 h with TNFα (100 ng/ml) in the presence/absence of the MEK_1/2_ inhibitor U0126 (10 μM) or ERK_1/2_ inhibitor SCH772984 (10 μM). After that, total RNA was prepared and used for real-time PCR measurement of the respective transcripts. Experimental groups denoted by different letters represent a significant difference at *P* < 0.05.

**Figure 7:**
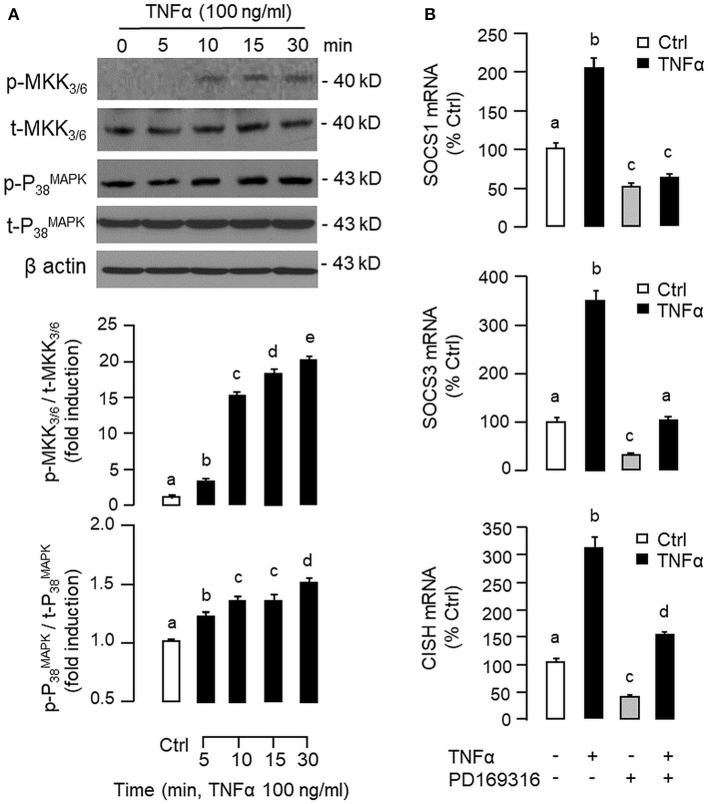
Functional role of MKK_3/6_/P_38_^MAPK^ cascade in TNFα-induced SOCS1, SOCS3, and CISH expression at the hepatic level. **(A)** TNFα treatment on MKK_3/6_ and P_38_^MAPK^ phosphorylation in carp hepatocytes. Hepatocytes were treated with TNFα (100 ng/ml) for the duration as indicated. After that, cell lysate was prepared and used in Western blot with antibodies for the phosphorylated form (p-form) and total protein (t-form) of MKK_3/6_ and P_38_^MAPK^, respectively, and parallel blotting of β actin was used as the internal control. **(B)** Blocking MKK_3/6_/P_38_^MAPK^ pathway on TNFα-induced SOCS1, SOCS3, and CISH mRNA expression. Hepatocytes were treated for 6 h with TNFα (100 ng/ml) in the presence/absence of the P_38_^MAPK^ inhibitor PD169316 (10 μM). After that, total RNA was isolated and used for real-time PCR measurement of the respective transcripts. Experimental groups denoted by different letters represent a significant difference at *P* < 0.05.

**Figure 8:**
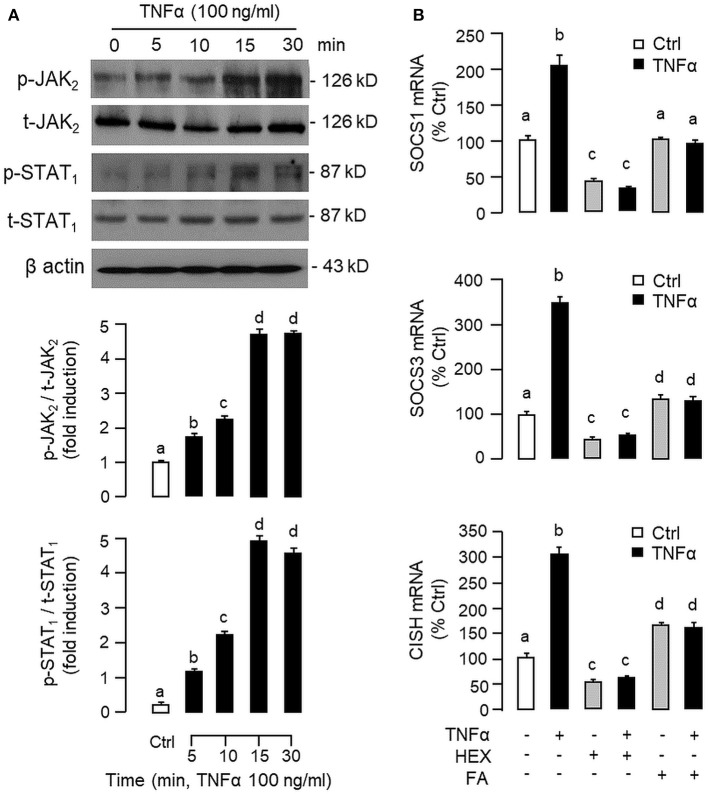
Functional role of JAK_2_ and STAT_1_ in TNFα -induced SOCS1, SOCS3, and CISH expression at the hepatic level. **(A)** TNFα treatment on JAK_2_ and STAT_1_ phosphorylation in carp hepatocytes. Hepatocytes were treated with TNFα (100 ng/ml) up to 30 min and cell lysate prepared at different time points was used in Western blot with antibodies for phosphorylated form (p-form) and total protein (t-form) of JAK_2_ and STAT_1_, respectively, and parallel blotting of β actin was used as the internal control. **(B)** Blocking JAK_2_ and STAT_1_ on TNFα-induced SOCS1, SOCS3, and CISH mRNA expression. Hepatocytes were treated for 6 h with TNFα (100 ng/ml) in the presence/absence of the JAK_2_ inhibitor HEX (50 μM) or STAT_1_ inhibitor FA (50 μM). After that, total RNA was isolated and used for real-time PCR measurement of the respective transcripts. Experimental groups denoted by different letters represent a significant difference at *P* < 0.05.

**Figure 9:**
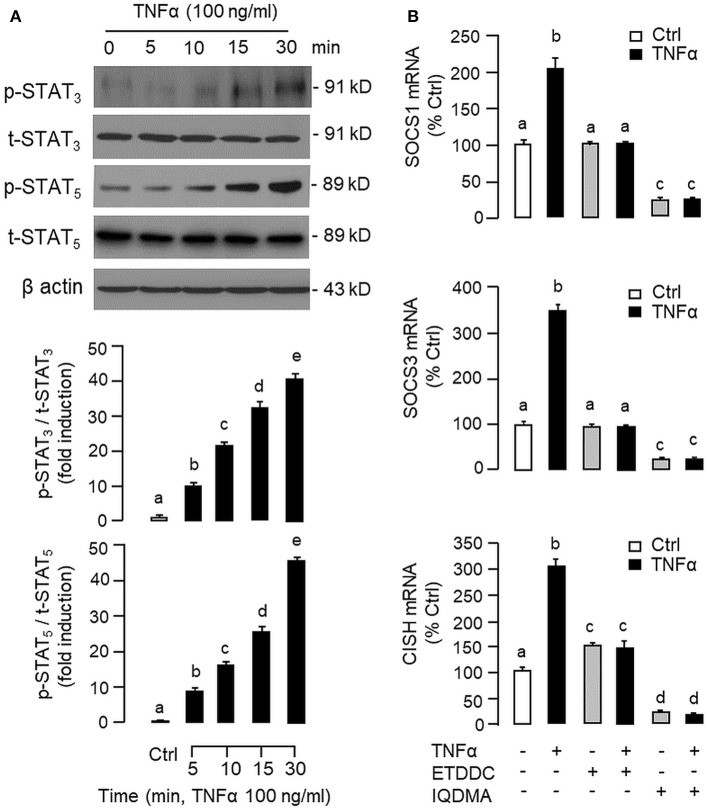
Functional role of STAT_3_ and STAT_5_ in TNFα-induced SOCS1, SOCS3, and CISH expression at the hepatic level. **(A)** TNFα treatment on STAT_3_ and STAT_5_ phosphorylation in carp hepatocytes. Hepatocytes were treated with TNFα (100 ng/ml) up to 30 min and cell lysate prepared was used in Western blot with antibodies for phosphorylated form (p-form) and total protein (t-form) of STAT_3_ and STAT_5_, respectively, and parallel blotting of β actin was used as the internal control. **(B)** Blocking STAT_3_ and STAT_5_ on TNFα-induced SOCS1, SOCS3, and CISH mRNA expression. Hepatocytes were treated for 6 h with TNFα (100 ng/ml) in the presence/absence of the STAT_3_ inhibitor ETDDC (300 nM) or STAT_5_ inhibitor IQDMA (50 μM). After that, total RNA was isolated and used for real-time PCR measurement of the respective transcripts. Experimental groups denoted by different letters represent a significant difference at *P* < 0.05.

**Figure 10:**
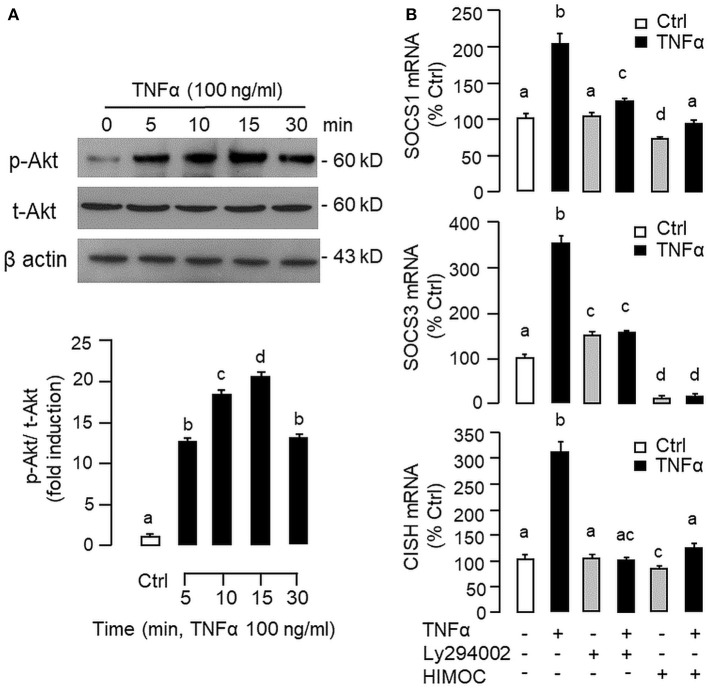
Functional role of PI3K/Akt cascade in TNFα-induced SOCS1, SOCS3, and CISH expression at hepatic level. **(A)** TNFα treatment on Akt phosphorylation in carp hepatocytes. Hepatocytes were treated with TNFα (100 ng/ml) up to 30 min and cell lysate prepared was used in Western blot with antibodies for phosphorylated form (p-form) and total protein (t-form) of Akt, and parallel blotting of β actin was used as the internal control. **(B)** Blocking PI3K/Akt cascade on TNFα-induced SOCS1, SOCS3, and CISH mRNA expression. Hepatocytes were treated for 6 h with TNFα (100 ng/ml) in the presence/absence of the PI3K inhibitor Ly294002 (10 μM) or Akt inhibitor HIMOC (10 μM). After that, total RNA was isolated and used for real-time PCR measurement of the respective transcripts. Experimental groups denoted by different letters represent a significant difference at *P* < 0.05.

To shed light on the post-receptor signaling mediating TNFα-induced SOCS1, SOCS3, and CISH expression at the hepatic level, Western blot was conducted to monitor the effects of TNFα on protein phosphorylation of various signaling targets in carp hepatocytes. In this case, short-term treatment with TNFα (100 ng/ml, up to 30 min) was found to be effective in triggering rapid phosphorylation of IκB (Figure 5A), MEK_1/2_ (Figure 6A), ERK_1/2_ (Figure 6A), MKK_3/6_ (Figure 7A), P_38_^MAPK^ (Figure 7A), JAK_2_ (Figure 8A), STAT_1_ (Figure 8A), STAT_3_ (Figure 9A), STAT_5_ (Figure 9A), and Akt (Figure 10A), respectively. Except for the drop in total content of IκB observed (Figure 5A), the total protein for other signaling targets examined did not show noticeable changes after TNFα treatment (Figures 6A–10A). In parallel studies, SOCS1, SOCS3, and CISH mRNA expression in carp hepatocytes were consistently up-regulated by TNFα (100 ng/ml) and these stimulatory effects could be reduced/abolished by co-treatment with the IKK inhibitor IKK16 (Figure 5B), NFκB inhibitor MG132 (Figure 5B), MEK_1/2_ inhibitor U0126 (Figure 6B), ERK_1/2_ inhibitor SCH 772984 (Figure 6B), P_38_^MAPK^ inhibitor PD169316 (Figure 7B), JAK_2_ inhibitor HEX (Figure 8B), STAT_1_ inhibitor FA (Figure 8B), STAT_3_ inhibitor ETDDC (Figure 9B), STAT_5_ inhibitor IQDMA (Figure 9B), PI3K inhibitor Ly294002 (Figure 10B), and Akt inhibitor HIMOC (Figure 10B), respectively. Besides, basal levels of SOCS1, SOCS3, and CISH transcript expression were also suppressed by the NFκB inhibitor MG132 (Figure 5B), MEK_1/2_ inhibitor U0126 (Figure 6B), P_38_^MAPK^ inhibitor PD169316 (Figure 7B), JAK_2_ inhibitor HEX (Figure 8B), STAT_5_ inhibitor IQDMA (Figure 9B), and Akt inhibitor HIMOC (Figure 10B).

## Discussion

As a first step to examine TNFα-induced GH resistance in the carp liver, the structural identity of grass carp TNFα was established by 5′/3′RACE. Based on phylogenetic analysis, the full-length cDNA obtained could be clustered in the clade of fish TNFα and closely related to the corresponding sequences in silver carp and common carp, the other members of the carp family. In fish models, two isoforms of TNFα encoded in separate genes are commonly reported in tetraploid species, including rainbow trout (49), common carp (50), and goldfish (51), which is assumed to be the result of the “fish-specific” (or 3R) whole genome duplication occurred prior to tetrapod evolution (52). In our study, a single form of TNFα was cloned and confirmed to be a single-copy gene in the carp genome, which is consistent with the fact that grass carp is a diploid fish, and presumably, had been branched off without the 3R genome duplication of the teleost lineage (53). The ORF of grass carp TNFα encodes a 239 a.a. protein with the 14 a.a. signature motif of the TNF family, a highly conserved transmembrane domain, and the typical feature of 12 antiparallel β sheets arranged in a “β-jellyroll” topography. The size of grass carp TNFα is similar to that of mammals, e.g., 233 a.a for human TNFα (54) and 235 a.a. for mouse TNFα (55), but notably smaller than that of the gilthead seabream (253 a.a.), flounder (256 a.a.), and rainbow trout (246 a.a.) (56). *In silico* protein modeling also reveals that, except for the lack of 2 short helixes in the linker between β sheet 6 and 7, the 3D structure of carp TNFα, especially for the spatial arrangement and orientation of the β sheets forming the compact jellyroll structure, is highly comparable to its human counterpart. Apparently, despite the a.a. substitutions occurred at protein level from fish to mammals, the 3D structure of TNFα is well conserved during vertebrate evolution, which may account for the bioactivity of human TNFα observed in immune cells of fish origin reported previously, e.g., inducing respiratory burst in macrophages (47) and cell proliferation in leucocytes in rainbow trout (46). It is also worth mentioning that five ARE elements could be located among the multiple polyadenylation signals within the 3′UTR of grass carp TNFα. These AU-rich cis-acting elements are commonly found in 3′UTR of “unstable transcripts” and serve as the binding sites for ARE-binding proteins, including TTP, KSRP, and AUF1, which are known to trigger mRNA deadenylation and exonucleolytic decay (57). Presence of multiple ARE elements in the 3′UTR of TNFα has also been reported in rainbow trout (49), catfish (58), goldfish (51), and flounder (59), implying that the TNFα transcript in fish models may not be very stable, and probably, has a fast turn-over rate, which is in agreement with the transient nature of pro-inflammatory responses mediated by TNFα (60).

In mammals, TNFα is expressed in immune cells including macrophages, monocytes, lymphocytes and neutrophils, especially on exposure to the bacterial endotoxin LPS. Similar findings have also been reported in fish models, e.g., in carp species (61, 62). Of note, TNFα expression can also be detected in non-immune cells/tissues, e.g., in astrocytes, microglia, fibroblast, and smooth muscle [for review, see (63)]. In rainbow trout (64), goldfish (51), tuna (65), and catfish (58), constitutive expression of TNFα has been reported in various tissues, especially in immune organs (e.g., thymus and head kidney) as well as in tissues with high chance of exposure to microbes (e.g., gills and intestine). In grass carp, as revealed by RT-PCR, high levels of TNFα signal were detected in the thymus, pituitary, and gonad, to a lower extent in the brain, spleen, and liver, but not in other tissues examined. Within the brain, low levels of TNFα signals were also located in the olfactory bulb, hypothalamus, optic tectum and medulla oblongata but not in other brain areas. In contrast to the wide spread/ubiquitous pattern of expression reported in other fish species, grass carp tends to have a tissue-specific pattern of TNFα expression. Although TNFα signal could be detected in the carp thymus, it was not found in the gills and head kidney, which are the major sites of TNFα expression in other fish models. The cause of the discrepancy is unclear and may be related to species-specific variations/culture environment of the species concerned. Apparently, basal expression of TNFα in most of the tissues/brain areas examined in grass carp (i.e., without experimentally induced infection/inflammation) is rather low/undetectable. Of note, our study also demonstrated for the first time that high levels of TNFα signals could be identified in the carp pituitary and gonad. The relevance of these findings related to endocrine regulation and reproductive functions is still unclear and future investigations are clearly warranted.

In our study with grass carp, detectable levels of TNFα transcript (by RT-PCR) and protein signals (by LC/MS/MS) could be identified in the liver, which is consistent with the idea that the liver can serve as a major immune target (e.g., for hepatitis virus) (66) as well as an immunological organ in vertebrates (e.g., as a source of complement factors and immune cells) (67). In primary culture of carp hepatocytes, LPS induction could trigger a transient rise in TNFα gene expression with parallel elevations in SOCS1, SOCS2, SOCS3, and CISH mRNA levels. A significant rise in TNFα signal was found to occur prior to those of type II SOCS and a gradual drop in TNFα response was also noted after the peak responses of SOCS1, SOCS2, SOCS3, and CISH expression. These findings corroborate with the idea that SOCS expression induced by infection/inflammation can serve as feedback inhibitors for cytokine signaling (17, 23). Of note, TNFα treatment was effective in mimicking the stimulatory effects of LPS on SOCS1, SOCS3, and CISH but not SOCS2 mRNA expression. In parallel experiments, transcript expression of IGF-I and -II induced by GH could be blocked by co-treatment with LPS and these inhibitory actions were also mimicked by TNFα and partially recovered by TNFα receptor antagonism. These findings, as a whole, suggest that (i) a functional system composed of TNFα and type II SOCS signals is present in the carp liver and inducible by endotoxin, presumably forming an integral component of the innate immunity in carp species, and (ii) type II SOCS expression induced by LPS at hepatic level, including SOCS1, SOCS3, and CISH, could be mediated by local production of TNFα, which may contribute to GH resistance for IGF-I/-II expression in the carp liver induced by endotoxin. In fish models including rainbow trout (64), catfish (58), and common carp (61, 62), TNFα expression induced by LPS has been reported, e.g., in macrophages and leucocytes, but the functional relevance of the phenomenon in GH resistance has not been examined. In mammals, endotoxin exposure can induce GH resistance at tissue level, e.g., in the liver, muscle and intestine (3, 4, 68). The effect is mediated by local production of cytokines including TNFα, IL-1β, and IL-6 (5, 68), which are known to reduce GH responsiveness by reducing GHR expression (10, 11) or blocking GHR signaling via SOCS expression (3, 13). Apparently, different cytokines have their distinct role in GH resistance induced by endotoxin (33). In liver cells or hepatoma cell lines (e.g., Huh-7 cells), TNFα and IL-1β induced by LPS only have minor effects on SOCS expression and their effects on GH resistance are mediated by reducing GHR gene transcription (32, 33), probably through inhibition of Sp1/3 binding to GHR promoter (10). In contrast, IL-6 signal induced by LPS has no effect on GHR expression but can serve as a potent stimulator for SOCS3 and CISH expression in these cell models (33, 69). In our recent study, type II SOCS including SOSC1-3 and CISH were up-regulated by GH in carp hepatocytes and over-expression of these SOCS members could inhibit JAK_2_/STAT_5_ signaling and block GH-induced IGF-I promoter activation, implying that type II SOCS can serve as feedback repressors for GH signing in carp species (41). In our current study with carp hepatocytes, GH resistance induced by LPS occurred with parallel rises in TNFα and type II SOCS signals with a concurrent drop in GHR expression. Unlike mammals, TNFα treatment did not alter GHR expression but was effective in increasing SOCS1, SOCS3, and CISH signals at the hepatic level. These results suggest that TNFα may exert its effect on GH resistance by “cross-inhibition” on GH signaling via SOCS expression to down-regulate IGF-I/-II responses in the carp liver.

Although TNFα-induced SOCS expression has been reported in different cell models, including the fibroblasts, macrophages and hepatocytes in rodents (32, 39), and TNFα receptors are well-documented to be functionally coupled to the IKK/NFκB, MAPK, PI3K/Akt, and JAK/STAT cascades (37, 38), the post-receptor signaling for TNFα-induced SOCS expression at the hepatic level is largely unknown. In carp hepatocytes, TNFα could induce a rapid phosphorylation of IκB, MEK_1/2_, ERK_1/2_, MKK_3/6_, P_38_^MAPK^, JAK_2_, STAT_1,3,5_, and Akt, respectively. Furthermore, pharmacological inhibition of IKK, NFκB, MEK_1/2_, ERK_1/2_, P_38_^MAPK^, PI3K, Akt, JAK_2_, and STAT_1,3,5_ were also effective in blocking SOCS1, SOCS3, and CISH mRNA expression induced by TNFα. These findings, as a whole, provide evidence that TNFα-induced type II SOCS expression in the carp liver (except for the lack of SOCS2 response) was mediated by the IKK/NFκB, MAPK, PI3K/Akt, and JAK/STAT cascades and the subsequent rises of SOCS/CISH signals presumably can play a role in TNFα-induced GH resistance at the hepatic level. In our study, the cellular content of IκB was reduced with concurrent rise in IκB phosphorylation after TNFα treatment. These findings imply that, similar to the mechanisms for NFκB activation in mammals (70), TNFα activation of IKK/NFκB pathway in carp liver may also involve IKK phosphorylation and proteosomal degradation of IκB to allow for nuclear translocation of NFκB and target gene transcription. In different cell models, TNFα activation of IKK/NFκB [e.g., IKK2 in hepatocytes (71)], MAPK [e.g., ERK_1/2_ in trophoblasts (72) and P_38_^MAPK^ in macrophages (39)], PI3K/Akt [e.g., Akt in myoblasts (73)], and JAK/STAT cascades [e.g., JAK_1_ in lymphoma B cells (74) and STAT_1,3,5_ in 3T3-L1 adipocytes (75)] have been reported and these post-receptor signaling pathways can also exhibit functional crosstalk at cellular level. For examples, TNFα-induced MMP-9 promoter activity via NFκB in HIPEC-65 cells is dependent on ERK_1/2_ activation (72). Previous studies on post-receptor signaling of TNFR1 in HEK293 cells reveal that MEKK1, the upstream activator of JNK_1/2_, can activate IKK with subsequent induction of IκB/NFκB cascade. Meanwhile, NFκB-inducing kinase (NIK) associated with TNFR1, an upstream activator of IKK, is also effective in cross-activation of JNK kinase, the upstream activator of P_38_^MAPK^ and JNK_1/2_ (76). In MCF-7 cancer cells, TNFα-induced NFκB activity is partly mediated by PI3K/Akt activation and NIK coupling to TNFR1 (77). Whether similar crosstalk in post-receptor signaling can also occur in carp hepatocytes and contribute to TNFα-induced GH resistance is unclear and remains to be elucidated.

**Figure 11:**
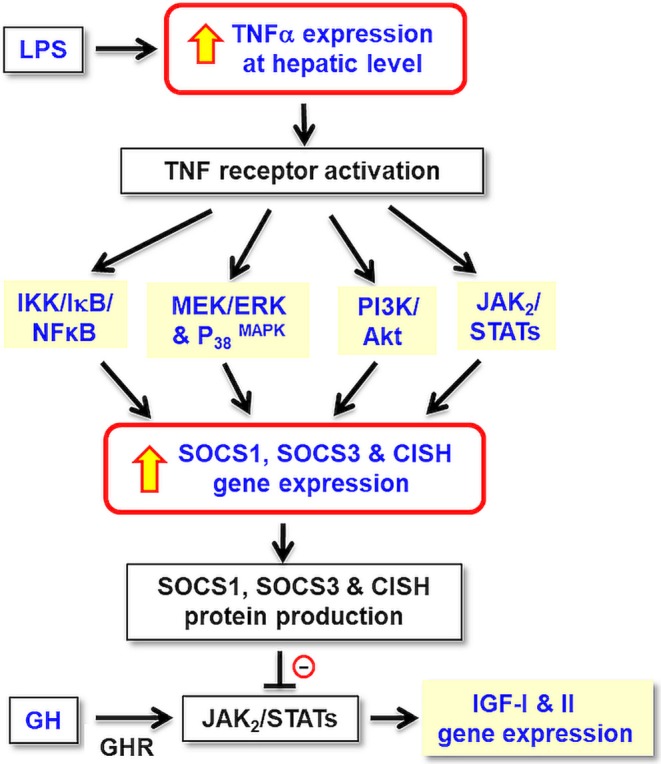
Working model for TNFα-induced SOCS expression in GH resistance caused by endotoxin in the liver of grass carp. In carp hepatocytes, LPS, the endotoxin in gram-negative bacteria, is effective in blocking GH-induced IGF-I and -II gene expression. These inhibitory actions are mediated by local production of TNFα followed by its induction of SOCS1, SOCS2, and CISH expression via the IKK/ NFκB, MAPK, PI3K/Akt, and JAK/STAT pathways. The subsequent rises in SOCS/CISH signals can suppress GH-induced IGF-I and -II expression probably by inhibiting the JAK_2_/STAT_5_ signing coupled to GHR in the carp liver.

In summary, grass carp TNFα was cloned and confirmed to be a single copy gene in the carp genome. Its tissue expression, including the liver, has been confirmed and functional studies in carp hepatocytes also reveal that TNFα together with its induction of type II SOCS, including SOCS1, SOCS3, and CISH, may constitute a local immune response induced by LPS and exert negative effects on GH-induced IGF -I and -II expression at hepatic level (Figure 11). Apparently, the stimulatory actions of TNFα on SOCS1, SOCS3, and CISH expression in carp hepatocytes are mediated via activation of IKK/NFκB, MAPK, PI3K/Akt, and JAK/STAT cascades. Our studies, as a whole, have shed light on the functional crosstalk between the immune system and somatotropic axis at the hepatic level in a fish model. In this case, local production of TNFα with subsequent induction of different SOCS members probably can mediate GH resistance commonly observed in the carp liver after infection with microbes/exposure to endotoxin. In carp hepatocytes, LPS was shown to elevate SOCS2 but suppress GHR expression and TNFα treatment was not effective in these regards. The discrepancy observed raises the possibility that other cytokines can also be induced by endotoxin exposure and contribute to GH resistance by functional coupling with SOCS2 and GHR regulation. In trout hepatocytes, cytokine expression induced by LPS (e.g., IL-8) can be blocked by cortisol co-treatment and cortisol alone is known to trigger SOCS1/2 up-regulation in the same model (78). Local production of other cytokines induced by LPS and their functional interactions with signals from the hypothalamo-pituitary-adrenal axis in GH resistance for sure can be an interesting topic for our future study in carp model.

## Data Availability Statement

The datasets generated for this study can be found in GenBank, at this link: https://www.ncbi.nlm.nih.gov/nuccore/JQ040498.

## Ethics Statement

The study was carried out according to the guidelines for the care and use of animals for research and teaching at the University of Hong Kong (Hong Kong). The protocol used in our study was approved by the Committee on the Use of Live Animal for Teaching and Research, University of Hong Kong.

## Author Contributions

AW was the PI and grant holder. AW and XJ were responsible for project planning and data analysis. XJ and JB were involved in molecular cloning of TNFα and functional studies in grass carp hepatocyte culture. MH was responsible for Western blot studies and LC/MS/MS detection of TNFα expression in the liver. Manuscript preparation was done by AW, CC, and XJ.

### Conflict of Interest

The authors declare that the research was conducted in the absence of any commercial or financial relationships that could be construed as a potential conflict of interest.

## Abbreviations

TNFα: Tumor necrosis factor α
SOCS: Suppressor of cytokine signaling
CISH: Cytokine-inducible SH2-containing protein
GH: Growth hormone
IGF: Insulin-like growth factor
LPS: Lipopolysaccharide
IKK: IκB kinase
NFκB: Nuclear factor kappa beta
MEK_1/2_: Mitogen-activated protein kinase kinase 1/2
ERK_1/2_: Extracellular signal-regulated kinase 1/2
P_38_^MAPK^: P_38_ Mitogen-activated protein kinase
PI3K: Phosphinositide 3-kinase
Akt
Protein kinase B
JAK_2_: Janus kinase 2
STAT: Signal transducer and activator of transcription.
