# Supplementary material for: Signal Transduction for TNFα-Induced Type II SOCS Expression and Its Functional Implication in Growth Hormone Resistance in Carp Hepatocytes
Source: Front Endocrinol (Lausanne). 2020 Jan 30;11:20. doi: 10.3389/fendo.2020.00020 (PMC7003395; doi:10.3389/fendo.2020.00020)
Supplement: Supplementary file 1 [file Data_Sheet_1.PDF]

Supplemental Table (1)

Primer sequences, PCR conditions and QC parameters for real-time PCR of target genes.

Gene name / Genbank accession no<br>Sequences of forward & reverse primers	Real-time PCR condition					Product size<br>& *Tm* value
	Denaturing	Annealing	Extension	Detection	Cycle no.	
SOCS1 / GU224284<br>Forward: 5'CAGCAGGCAGCCATTT 3'<br>Reverse: 5' GACCGTGTTGTCCTTTGT 3'	94 °C<br>30 sec	49°C<br>30 sec	72 °C<br>30 sec	80 °C<br>20 sec	×35	232 bp<br>(*Tm* = 83.1°C)
SOCS2 / GQ478990<br>Forward: 5' CGTCGGAGGGCACATTTC 3'<br>Reverse: 5' CGGAGTCGTGAGCAGCAG 3'	94 °C<br>30 sec	55°C<br>30 sec	72 °C<br>30 sec	82 °C<br>20 sec	×35	280 bp<br>(*Tm* =86.1 °C)
SOCS3/ EU625352<br>Forward: 5' GGTCCG AGATATGTTGAATGA 3'<br>Reverse: 5' CAGTGTCTTGTATGACAAGGTG 3'	94 °C<br>30 sec	55 °C<br>30 sec	72 °C<br>30 sec	82 °C<br>20 sec	×35	240 bp<br>(*Tm* = 84.8 °C)
CISH / GU384205<br>Forward: 5' AGAGCTCATTTGCTGATACCT 3'<br>Reverse: 5' GGAAACGTCCGAGACTGTACT 3'	94 °C<br>30 sec	62 °C<br>30 sec	72 °C<br>30 sec	84 °C<br>20 sec	×35	200 bp<br>(*Tm* = 85.2 °C)
TNFα /JQ040498<br>Forward: 5' GCTTCACGCTCAACAAGTCTCA 3'<br>Reverse: 5' AGCCTGGTCCTGGTTCACTCT 3'	94 °C<br>30 sec	56 °C<br>30 sec	72 °C<br>30 sec	84 °C<br>20 sec	×35	179 bp<br>(*Tm* = 85 °C)
GHR / AY283778.2<br>Forward: 5' CCTTGGCGTCCAGCAGACATC 3'<br>Reverse: 5' TACTCCCCATTCCAGAGGTTCC 3'	94 °C<br>30 sec	70 °C<br>30 sec	72 °C<br>30 sec	81 °C<br>20 sec	×35	352 bp<br>(*Tm* = 85.2 °C)
IGF-I/ AF247658.1<br>Forward: 5' TCTCACTGGTGCTGTGCGTCCTCGCG 3'<br>Reverse: 5' GCTCTGAAAGCAGCATTCGTCCACAA 3'	94 °C<br>30 sec	65 °C<br>30 sec	72 °C<br>30 sec	84 °C<br>20 sec	×35	203 bp<br>(*Tm* = 87.5 °C)
IGF-II/ EF062860.1<br>Forward: 5' CGTGGGATCGTGGAGATTTG 3'<br>Reverse: 5' TGGGACCTCCTGTTTTAATGC 3'	94 °C<br>30 sec	62 °C<br>30 sec	72 °C<br>30 sec	84 °C<br>20 sec	×35	150 bp<br>(*Tm* = 88 °C)
18S RNA / HQ615531.1<br>Forward: 5' AGCAACTTTAGTATACGCTAT 3'<br>Reverse: 5' CTGAGAAACGGCTACCACATC 3'	94 °C<br>30 sec	64°C<br>30 sec	72 °C<br>30 sec	79 °C<br>20 sec	×30	210 bp<br>(*Tm* = 85°C)
