# Supplementary material for: Signal Transduction for TNFα-Induced Type II SOCS Expression and Its Functional Implication in Growth Hormone Resistance in Carp Hepatocytes
Source: Front Endocrinol (Lausanne). 2020 Jan 30;11:20. doi: 10.3389/fendo.2020.00020 (PMC7003395; doi:10.3389/fendo.2020.00020)
Supplement: Supplementary file 2 [file Image_1.pdf]

Supplemental Fig.1

```
1    ccagcaaagcactgaagtgataaactgcactgaggacacttacagaaccttttaaaaagcattttgacagcatct
76   gcattttacatatagtttaacttaagctttcagaggacaaacacacagta ATG ATG GAG CAT GCC AGT
1                                                        M   M   E   H   A   S
145  CAG GTA GTG TTG GAT CTT GAA AAA GTA ACG CTG CCC TTG CCG AGG GTG ATG GTG CCG
7     Q   V   V   L   D   L   E   K   V   T   L   P   L   P   R   V   M   V   P
202  AGG AGG AAG GCT GGC ACT TCA AAG TCA GGC GTA TGG CGG GTG TGT GGG GCT CTT CTG
26    R   R   K   A   G   T   S   K   S   G   V   W   R   V   C   G   A   L   L
259  GCT GTG GCC CTG TGT GCT GCC GCT GCT GTC TGC TTC ACG CTC AAC AAG TCT CAG AGT
45    A   V   A   L   C   A   A   A   A   V   C   F   T   L   N   K   S   Q   S
316  AAT CAG GAA AGT GCA ACT GGG CTC AAG CTT ACA ATG AGA GAT CAT TTT TCA AAA GCA
64    N   Q   E   S   A   T   G   L   K   L   T   M   R   D   H   F   S   K   A
373  AAT TTC ACT TCC AAG GCT GCC ATC CAT TTA ACA GGT GCA TAC GAC CCT GAA GTC TCT
83    N   F   T   S   K   A   A   I   H   L   T   G   A   Y   D   P   E   V   S
430  AAT AAA ACC CTA GAT TGG AGA GTG AAC CAG GAC CAG GCT TTC TCT TCA GGC GGC TTG
102   N   K   T   L   D   W   R   V   N   Q   D   Q   A   F   S   S   G   G   L
487  AAA TTA GTA AAC AGG GAG ATC ATC ATT CCT GAT GAC GGC ATT TAC TTC GTC TAC AGC
121   K   L   V   N   R   E   I   I   I   P   D   D   G   I   Y   F   V   Y   S
544  CAG GTC TCT TTC CAC ATC TGC TGC GCG TCT GAC AGG GGC GCG GAC CAA GAC ATT GTG
140   Q   V   S   F   H   I   C   C   A   S   D   R   G   A   D   Q   D   I   V
601  CAT ATG AGC CAC GCA GTT ATG CGA ATT TCC GAT TCC TAT GGA GGC AAA AAG GCG CTT
159   H   M   S   H   A   V   M   R   I   S   D   S   Y   G   G   K   K   A   L
658  TTC AGC GCT ATC CGC TCC GCC TGC GTG CAC GCG TCT GAC AGT GAC GAT TTG TCG TAC
178   F   S   A   I   R   S   A   C   V   H   A   S   D   S   D   D   L   S   Y
715  AAC ACA ATT TAT CTC GGT GCG GCC TTC CAA TTA CAA GCT GGA GAC AAA CTG CTC ACC
197   N   T   I   Y   L   G   A   A   F   Q   L   Q   A   G   D   K   L   L   T
772  GAG ACG ACG CCA CTA CTC CTG CCC CGC GTC GAA AAT GAA AAC GGA AAG ACC TTT TTT
216   E   T   T   P   L   L   L   P   R   V   E   N   E   N   G   K   T   F   F
829  GGG  GTG TTT GCT CTG TAA gcatagatggacatcaagcaaaacttgaaaagtaaggaaagaatggaaac
235   G    V   F   A   L   *
897  ctgtggagaggaaagaaatttcctgagcttccactgcaatgtcacacacacaaaaagaaactaaactgacttcta
972  gccaacgttttatattaaaacaggtcaaaattataactctatttaatggatcagaaggatccagatatgatgtta
1047 tgtggggaaaacatgggcattttgtattcagctgtaaagctatcaaagttatttatatatgtttgttaaaatgt
1122 taatgttatgtgtatttattgtgtaattattttattgtactatttattcacctatttattaaagaatatctact
1179 gcactgcaactgtaacgaaattcaaattttaataaatctaaaaccaaatgaaaaa
```

Supplemental Fig.1. Nucleotide and protein sequences of grass carp TNFα. The full-length cDNA of grass carp TNFα was constructed using MacVector 6.5. The protein sequence deduced from the ORF are presented along with the corresponding cDNA sequences. The numbers on the left indicate the position of the first nucleotide at the beginning of individual lines for cDNA sequence. The ORF is presented in upper cases whereas the 5' and 3' UTR are presented in lower cases. The TNF family signature motif was boxed and transmembrane domain of TNFα was underlined in red. The TACE cleavage site ("TL") was labeled in red. The two cysteine residues essential for maintaining the tertiary structure of TNFα were circled and putative glycosylation sites were marked by pink dotted line. An asterisk (*) represents the stop codon located at the end of the ORF. In the 3'UTR, four ARE elements ("attta") and three polyadenylation signals ("aaaaag" and "attaaa") were identified and underlined in blue and black, respectively.
