# Supplementary material for: Signal Transduction for TNFα-Induced Type II SOCS Expression and Its Functional Implication in Growth Hormone Resistance in Carp Hepatocytes
Source: Front Endocrinol (Lausanne). 2020 Jan 30;11:20. doi: 10.3389/fendo.2020.00020 (PMC7003395; doi:10.3389/fendo.2020.00020)
Supplement: Supplementary file 3 [file Image_2.pdf]

Supplemental Fig.2

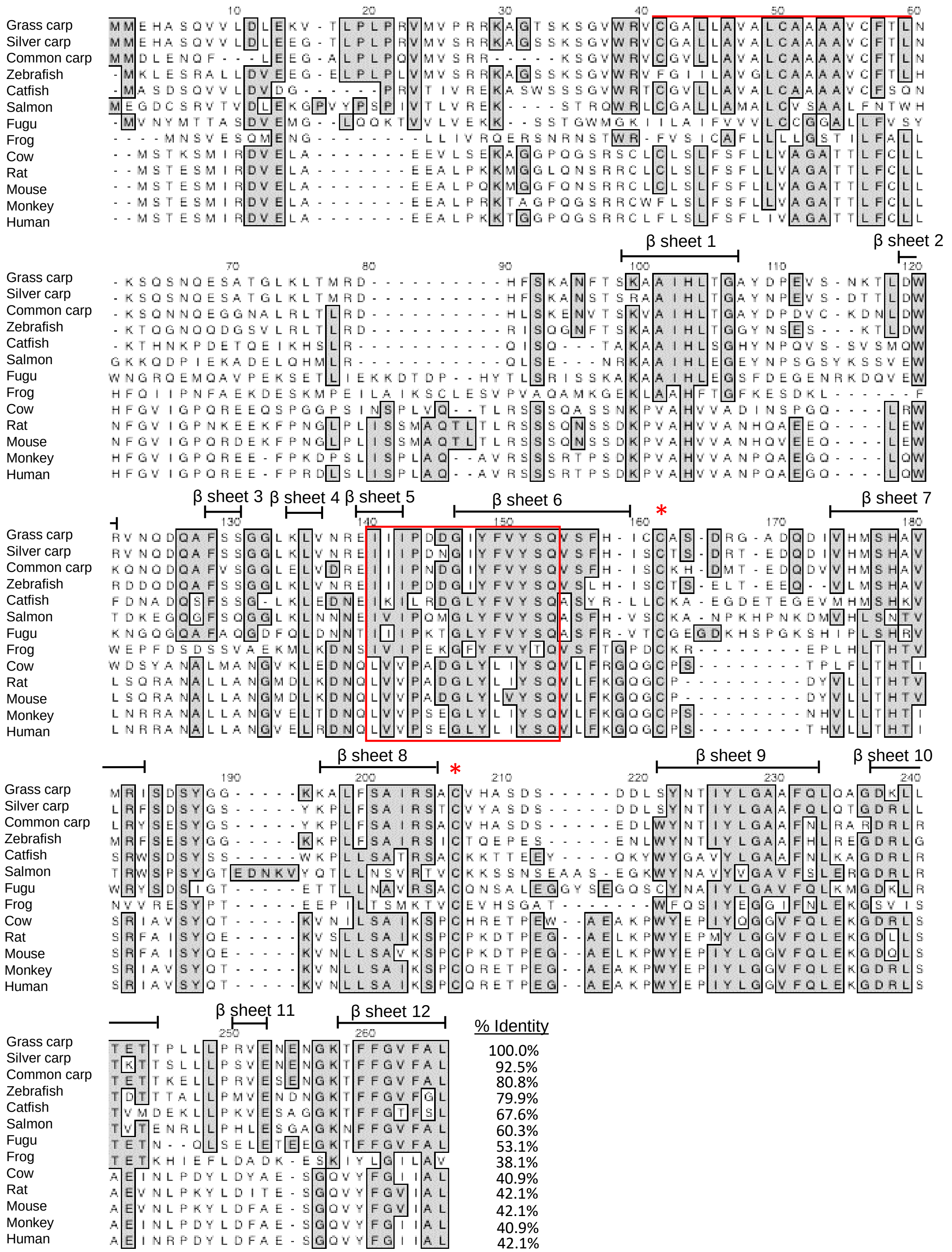

Supplemental Fig.2. Protein sequence alignment of carp TNFα with those reported in other vertebrates using Clustal W algorithm. The dashes within individual protein sequences represent the gaps inserted to maximize sequence alignment. The transmembrane domain deduced by TopPred II software is marked by a red line above the corresponding sequences while the TNF family signature motif is boxed in red. The two conserved cysteine residues essential for maintaining the tertiary structure of TNFα are marked by red asterisks. The regions covered by β sheet 1-12 are indicated by horizontal bars above the sequences presented.
