# Supplementary material for: Signal Transduction for TNFα-Induced Type II SOCS Expression and Its Functional Implication in Growth Hormone Resistance in Carp Hepatocytes
Source: Front Endocrinol (Lausanne). 2020 Jan 30;11:20. doi: 10.3389/fendo.2020.00020 (PMC7003395; doi:10.3389/fendo.2020.00020)
Supplement: Supplementary file 4 [file Image_3.pdf]

Supplemental Fig.3

(A)

Grass Carp TNFα (Protein coverage by peptides identified in B: 85.4%, grass carp liver)

Peptide 1

MMEHASQVVLDLEKVTLPLPRVMVPRRKAGTSKSGVWRVCGALLAVALCAAAAVCFTLNKSQSNQESATGLKLTMRDHFSKANF

Peptide 2

TSKAAIHLTGAYDPEVSNKTLDWRVNQDQAFSSGGLKLVNREIIIPDDGIYFVYSQVSFHICCASDRGADQDIVHMSHAVMRIS

Peptide 3

DSYGGKKALFSAIRSACVHASDSDDLSYNTIYLGAAFQLQAGDKLLTETTPLLLPRVENENGKTFFGVFAL

(B)

Peptides identified with 99% confidence

Conf	Peptide Sequence	ΔMass	Prec MW	z	Prec m/z
99	AAIHLTGAYDPEVSNK	-0.051	2293.2	4	574.3
99	AFSSGGLK	0.062	1069.7	2	535.8
99	AGTSK	0.056	1070.6	2	536.3
99	AGTSKSGVWR	-0.013	1655.9	3	553.0
99	AIHLTGAYDPEV	0.069	1588.9	3	530.6
99	ALFSAIR	-0.046	1080.6	2	541.3
99	ANFTSK	-0.043	1275.7	2	638.8
99	ANFTSKAAIHLTGAYDPEVSNK	-0.148	3245.6	4	812.4
99	DHFSK	-0.048	1240.7	2	621.4
99	DHFSKANFTSK	-0.104	2193.1	4	549.3
99	GAAFQLQ	0.107	1037.7	2	519.9
99	GADQDIVHMSH	0.099	1512.8	3	505.3
99	GADQDIVHMSHAVMR	-0.004	1970.0	2	986.0
99	GADQDIVHMSHAVMRISDSYGGK	0.027	3081.6	4	771.4
99	ISDSYGGK	-0.082	1433.8	2	717.9
99	ISDSYGGKK	-0.014	1866.0	3	623.0
99	ISDSYGGKKALFSAIR	-0.141	2624.4	3	875.8
99	LLPR	-0.008	801.5	2	401.8
99	LLTETTPLLLPRVENENGK	-0.135	2745.4	3	916.2
99	LPLPRVMV	0.000	1227.8	3	410.3
99	LTMR	0.024	839.5	2	420.8
99	LTMRDHFSK	-0.069	1741.9	2	872.0
99	LTMRDHFSKANFTSK	0.022	2710.5	3	904.5
99	MEHASQVVLDLEK	0.021	2107.2	3	703.4
99	MMEHASQVVLDLEK	-0.056	2065.1	3	689.4
99	SGVWRVCGALLAVALCAAAAVCFTLNK	-0.013	3452.8	6	576.5
99	SQSNQESATGLK	-0.019	1858.0	3	620.4
99	SQSNQESATGLKLTMRDHFSK	-0.034	3277.7	4	820.4
99	TFFGVFAL	0.010	1204.7	3	402.6
99	TLDWR	0.017	993.6	2	497.8
99	TLDWRVNQDQAFSSGGLK	-0.066	2629.3	3	877.4
99	VCGALLAVALCAAAAVCFTLNK	-0.014	2867.5	4	717.9
99	VENENGK	0.057	1397.8	3	466.9
99	VENENGKTFFGVFAL	0.016	2279.3	3	760.8
99	VMVPR	-0.020	904.6	2	453.3
99	VMVPRR	0.007	1076.7	2	539.3
99	VMVPRRK	-0.123	1492.8	3	498.6
99	VNQDQAFSSGGLK	0.002	1958.2	4	490.5
99	VNQDQAFSSGGLKLVNR	0.049	2440.4	3	814.5
99	VTLPLPRVMVPR	0.041	1697.1	2	849.5
99	VTLPLPRVMVPRR	0.067	1837.1	3	613.4
99	IIPDDGIYFVYSQV	0.172	1936.1	3	646.4

(C)

Representative mass spectra

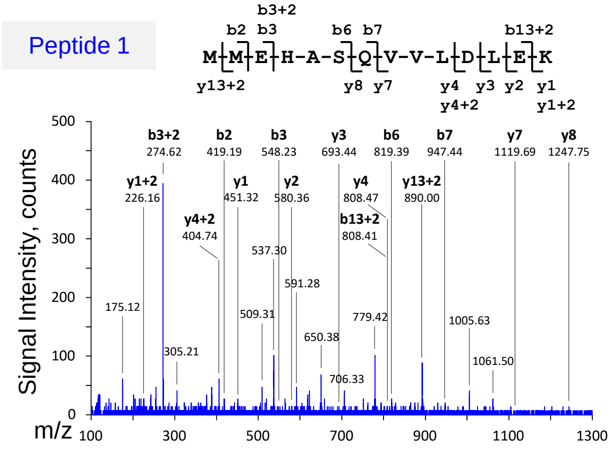

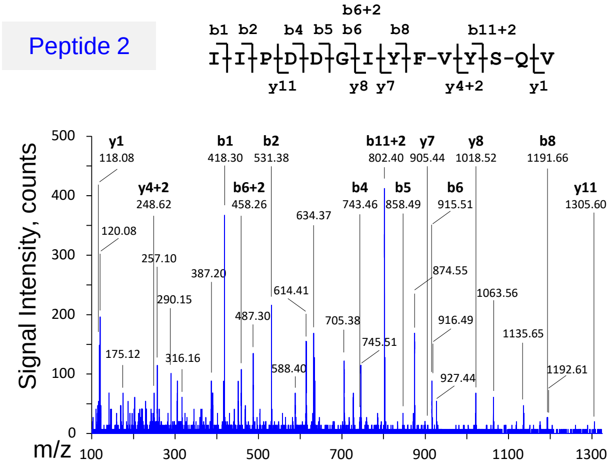

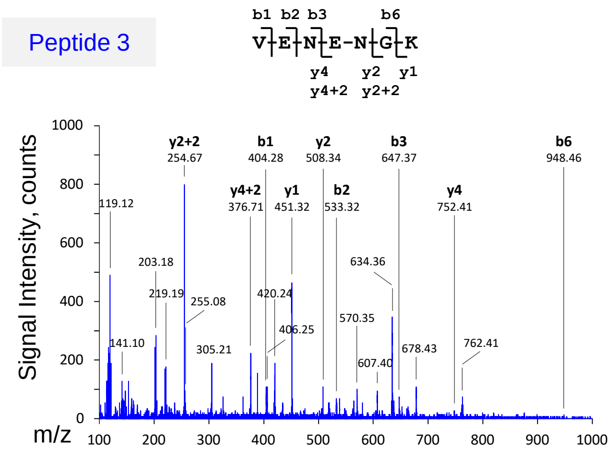

Supplemental Fig.3. Detection of protein expression of TNFα in the carp liver using proteomic approach. Protein lysate was prepared from the carp liver and subjected to trypsin digestion followed by LC/MS/MS. The digested products were resolved by C_{18} chromatography followed by MS/MS detection. Peptide products originated from TNFα were identified by ProteinPilot 2.0 based on the a.a. sequence of carp TNFα presented. For MS/MS data presented, locations of the peptides identified with confidence level at 99% were mapped (by underlines) in the a.a. sequence of carp TNFα presented in (A). The sequences of the peptides identified together with the corresponding QC data, including the percentage of confidence (as "Confid.") and mass derivation (as "ΔMass"), and MS data, including precise molecular weight (as "Prec MW"), theoretical charge (as "Z") and precise mass-to-charge ratio (as "Prec m/z"), are presented in (B). Representative MS/MS spectra of peptides originated from TNFα, including peptide 1, 2 and 3 (shaded in grey within the protein sequence of carp TNFα), are presented in (C). For MS/MS spectra, the ion peaks corresponding to the series of b- and y-fragments generated by collision-induced fragmentation have been annotated for the respective peptide targets.
